# Supplementary figures and images for: Shade suppresses wound-induced leaf repositioning through a mechanism involving PHYTOCHROME KINASE SUBSTRATE (PKS) genes
Source: PLoS Genet. 2022 May 27;18(5):e1010213. doi: 10.1371/journal.pgen.1010213 (PMC9197076; doi:10.1371/journal.pgen.1010213)

A

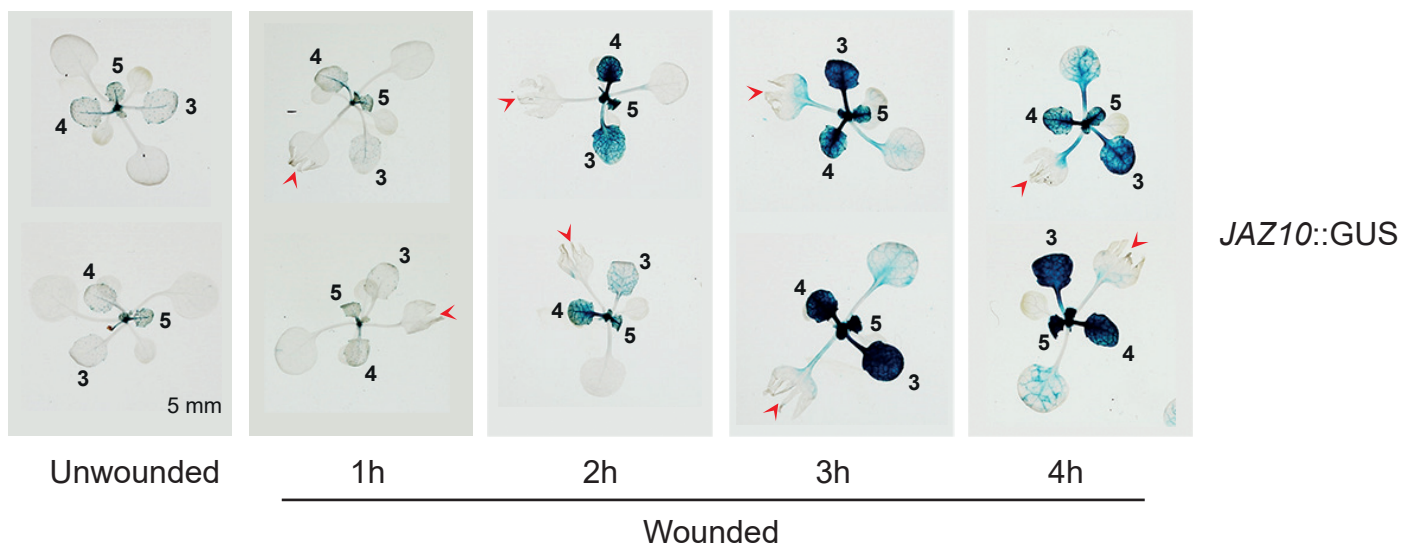

B

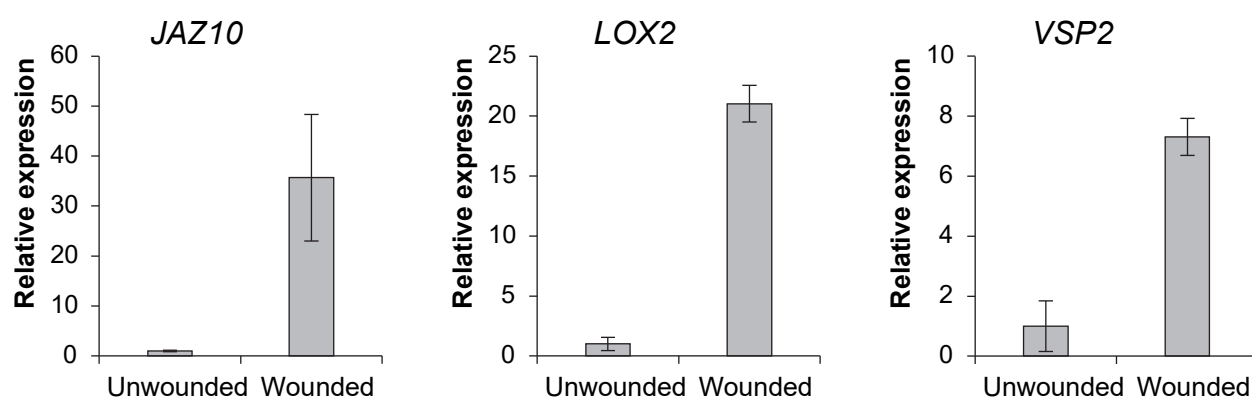

C

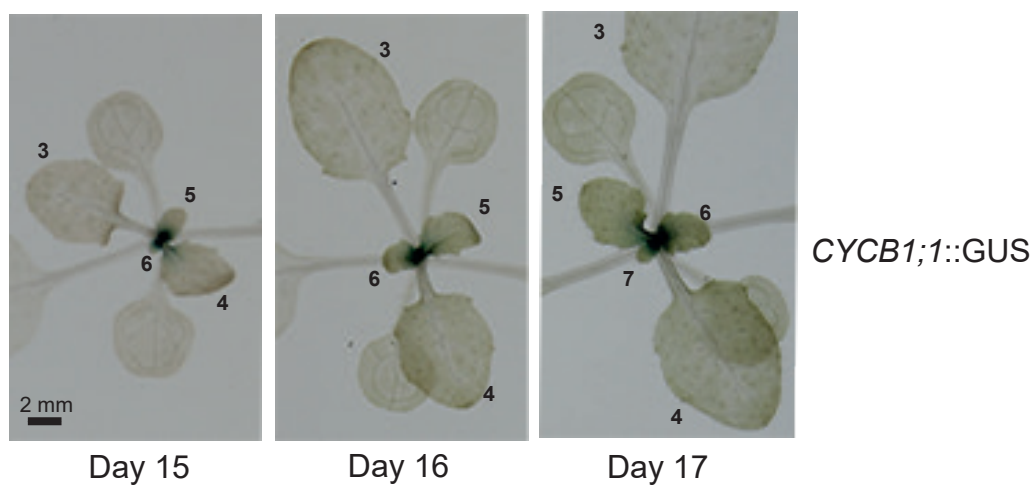

Supplement: S1 Fig — A) Time-course GUS activity in rosettes of the JAZ10:GUS reporter line upon wounding of leaf 1. 15-day-old rosettes grown in long days under high R/FR conditions were harvested at different times after wounding. Red arrow: wounded leaf. B) RT-qPCR analysis of JA-induced genes (JAZ10, LOX2, VSP2) in entire leaf 4 of unwounded and wounded JAZ10:GUS plants. 16-day-old rosettes grown in LD, high R/FR, were either touched (control, unwounded) or wounded on leaf 1 at ZT3 and leaves 4 were harvested 3h after treatment. For each gene, expression levels are given relative to housekeeping genes and expression in unwounded plants is arbitrarily set to 1. Error bars correspond to SD from 3 biological replicates. C) GUS activity in the CYCB1;1:GUS reporter line as a marker for dividing cells. Rosettes grown in long days under high R/FR conditions were harvested from day 15 to 17 after sowing. Leaves are numbered from the oldest to the youngest. Scale bar, 2 mm. (PDF) [file pgen.1010213.s005.pdf]

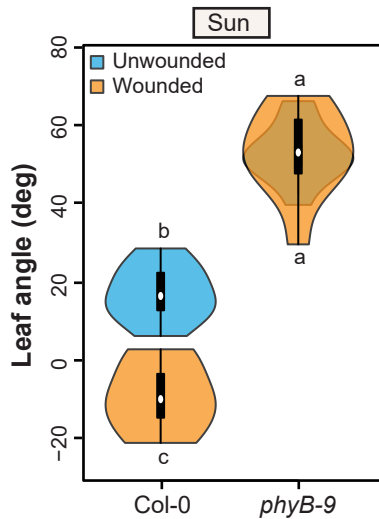

ANOVA  
Wound x Genotype:  
pval =  $1.89 \times 10^{-5}$

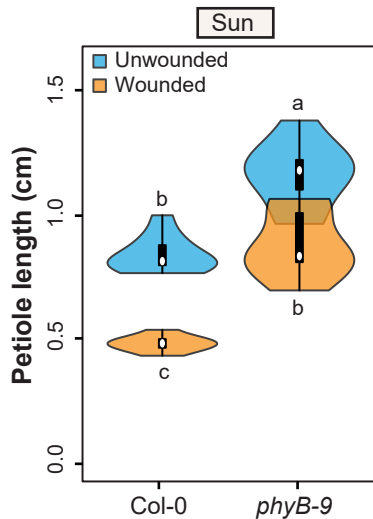

ANOVA  
Wound x Genotype:  
Not significant

Supplement: S3 Fig — Leaf angle and petiole length phenotype of wt Col-0 and phyB-9 mutant plants grown in high R/FR (n = 10 plants/genotype/condition). As described on Fig 1A, wounds are performed individually on leaf 1 (day 16), then leaf 2, then leaf 3 and plants are then kept in sun condition. We measured elevation angle of leaf 4 at the end of day 18 on pictures of each individual plant and petiole length of the same leaf at the end of the treatments (day 21). Representative experiment from 3 biological replicates. Graphs are represented as violin plots, which present a combination of a box plot and a kernel density plot. In each box plot, the white dot represents the median, black boxes extend from the 25th to the 75th percentile, while the vertical black line extends to 1.5 times the interquartile range of the lower and upper quartiles, respectively. A rotated kernel density plot surrounds each side of the box plot. Different letters indicate significant differences (Tukey’s HSD test following a two-way ANOVA, P < 0.05). (PDF) [file pgen.1010213.s007.pdf]

A

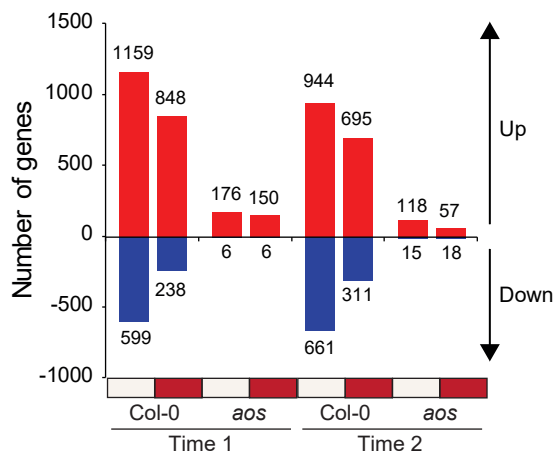

B

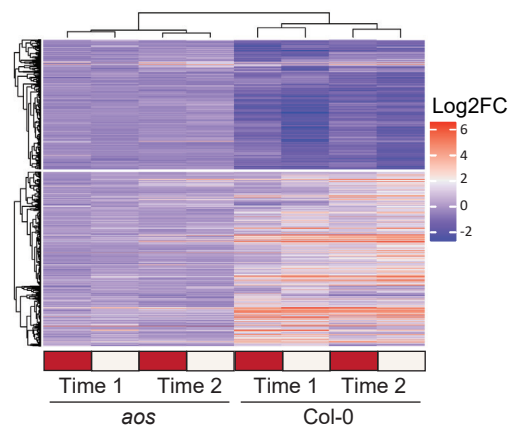

C

All wound-regulated genes (at Time 1 and/or Time 2)

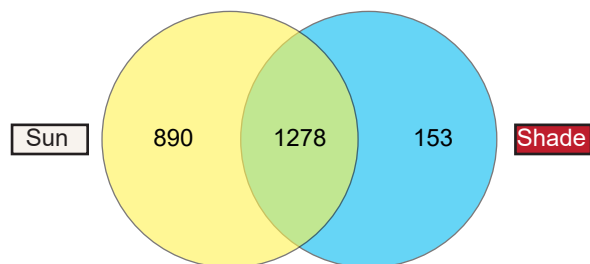

D

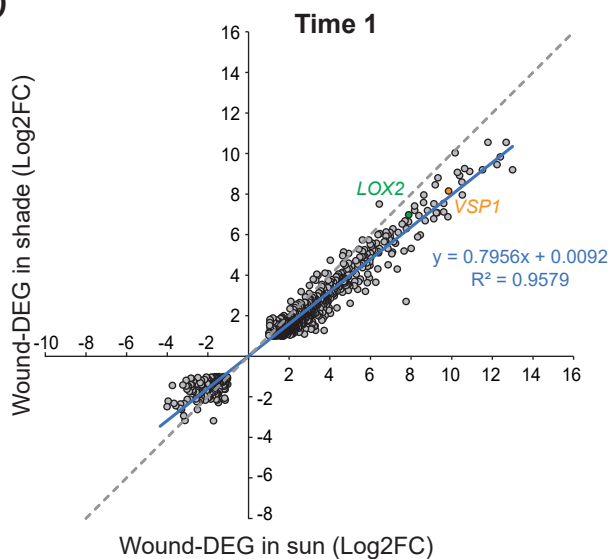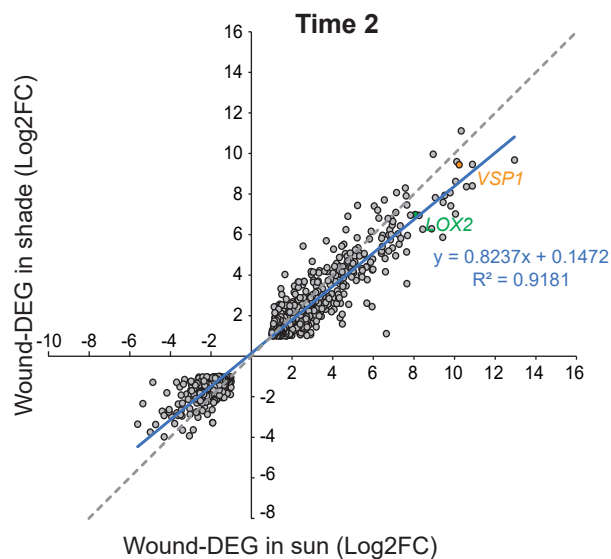

Supplement: S4 Fig — A) Number of genes significantly differentially regulated by wounding in the different conditions: genotypes (Col-0 and aos), timepoints (Time 1 and Time 2) and shade treatment (sun in beige, shade in dark red). Genes are considered differentially expressed when |log2 fold change|>1 and pval < 0.05. B) Heatmap with hierarchical clustering of significantly differentially regulated genes by wounding in the different conditions (same genes as in A). C) Venn diagram comparing wound-regulated genes (at Time 1 and/or Time 2) in sun vs shade. D) Scatterplots of expression (Log2 FC) of wound-regulated genes in sun vs shade at Time 1 (left graph) or Time 2 (right graph). Blue line represents the linear regression of shown data points (equation and regression coefficient are added on the right). Dotted grey line represents y = x. (PDF) [file pgen.1010213.s008.pdf]

A

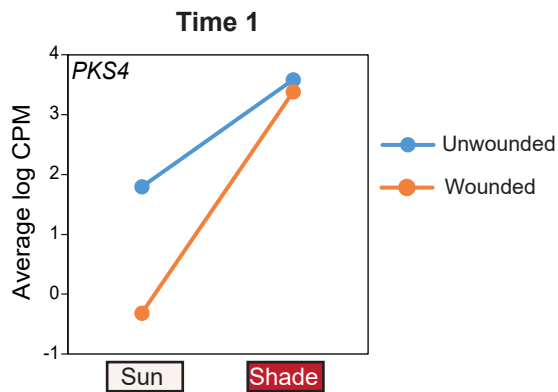

B

*PKS4::GUS*

Unwounded

Sun

Shade

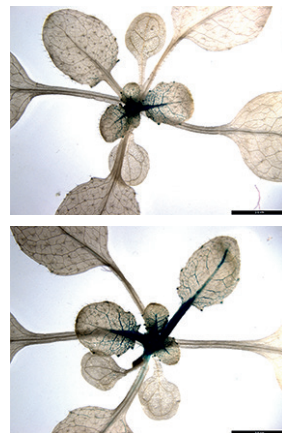

C

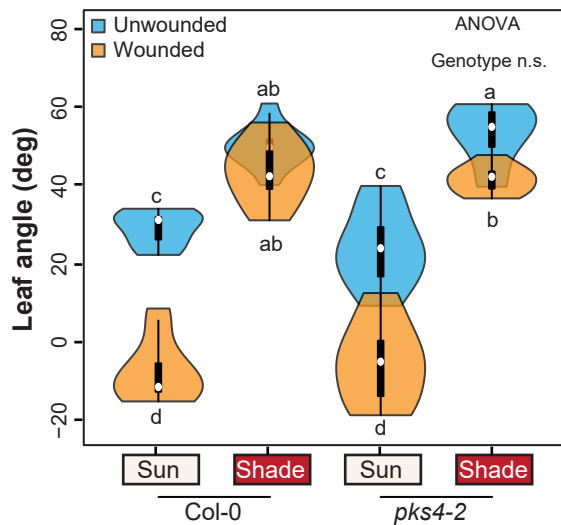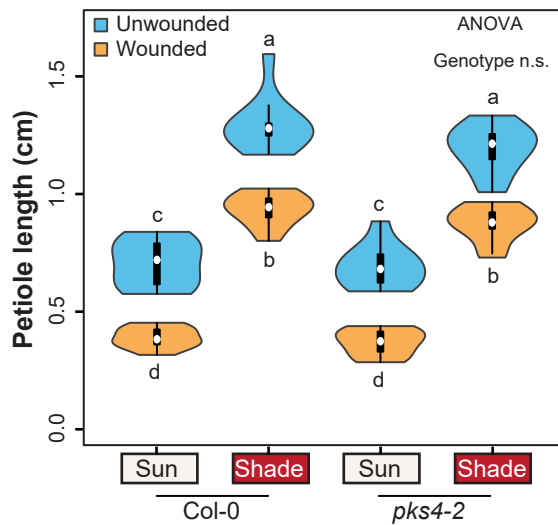

Supplement: S5 Fig — A) PKS4 expression in RNA-seq dataset (at Time 1, 1h30, wt samples). Values correspond to average log CPM from the three biological replicates. B) PKS4 expression in PKS4::GUS reporter line subjected to shade treatment. Rosettes grown in long days under high R/FR conditions were either kept in high R/FR or subjected to low R/FR for 6 hours starting at ZT3 and harvested on day 18 after sowing. Scale bar, 3.6 mm. C) Leaf angle and petiole length phenotype of wt Col-0 and pks4-2 mutant plants (n = 10 plants/genotype/condition). Representative experiment from 2 biological replicates. Experiments were done as described on Fig 1A. Graphs are represented as violin plots, which present a combination of a box plot and a kernel density plot. In each box plot, the white dot represents the median, black boxes extend from the 25th to the 75th percentile, while the vertical black line extends to 1.5 times the interquartile range of the lower and upper quartiles, respectively. A rotated kernel density plot surrounds each side of the box plot. Different letters indicate significant differences (Tukey’s HSD test following a three-way ANOVA, P < 0.05). (PDF) [file pgen.1010213.s009.pdf]

A

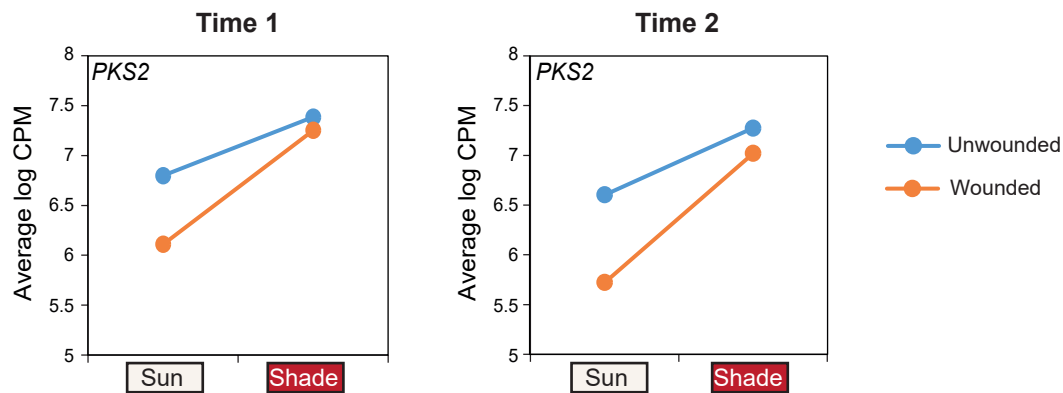

B

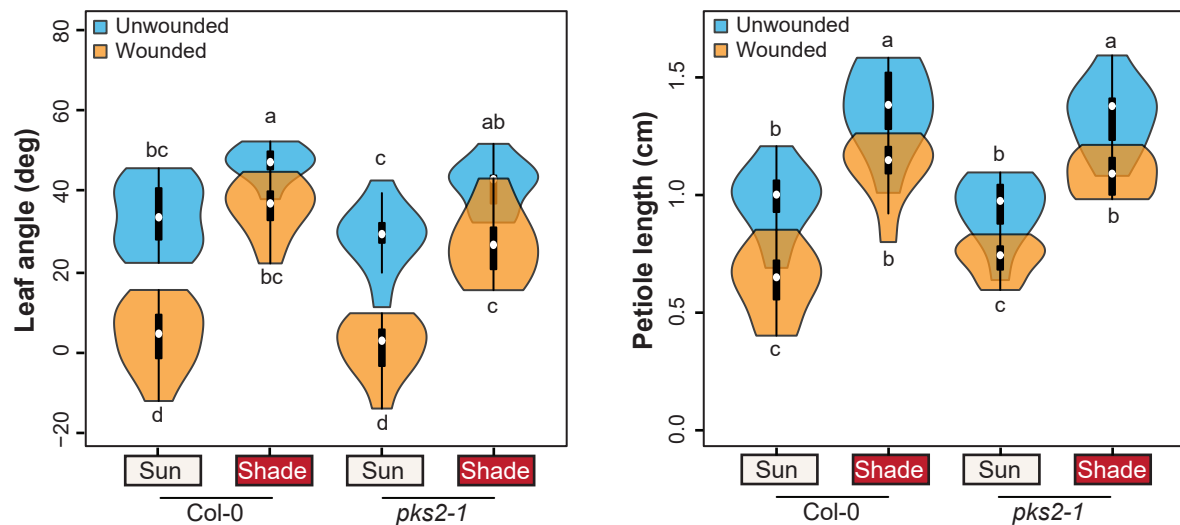

Supplement: S6 Fig — A) PKS2 expression in RNA-seq dataset (left at Time 1, right at Time 2, wt samples). Values correspond to average log CPM from the three biological replicates. B) Leaf angle and petiole length phenotype of wt Col-0 and pks2-1 mutant plants (n = 10 plants/genotype/condition). Representative experiment from 2 biological replicates. Experiments were done as described on Fig 1A. Graphs are represented as violin plots, which present a combination of a box plot and a kernel density plot. In each box plot, the white dot represents the median, black boxes extend from the 25th to the 75th percentile, while the vertical black line extends to 1.5 times the interquartile range of the lower and upper quartiles, respectively. A rotated kernel density plot surrounds each side of the box plot. Different letters indicate significant differences (Tukey’s HSD test following a three-way ANOVA, P < 0.05). (PDF) [file pgen.1010213.s010.pdf]

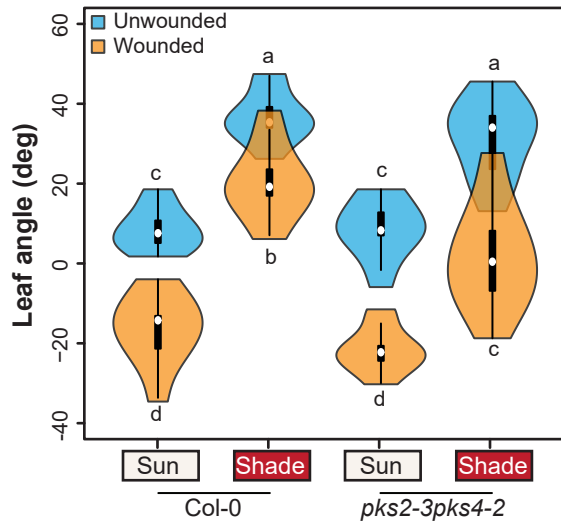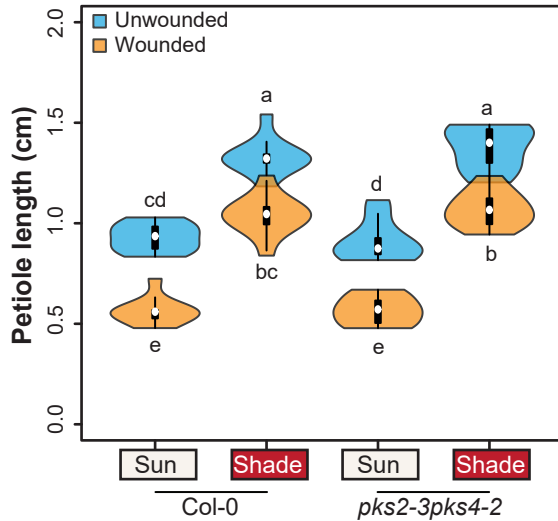

Supplement: S7 Fig — Leaf angle and petiole length phenotype of wt Col-0 and pks2-3pks4-2 mutant plants (n = 10 plants/genotype/condition). Representative experiment from 2 biological replicates. Experiments were done as described on Fig 1A. Graphs are represented as violin plots, which present a combination of a box plot and a kernel density plot. In each box plot, the white dot represents the median, black boxes extend from the 25th to the 75th percentile, while the vertical black line extends to 1.5 times the interquartile range of the lower and upper quartiles, respectively. A rotated kernel density plot surrounds each side of the box plot. Different letters indicate significant differences (Tukey’s HSD test following a three-way ANOVA, P < 0.05). (PDF) [file pgen.1010213.s011.pdf]

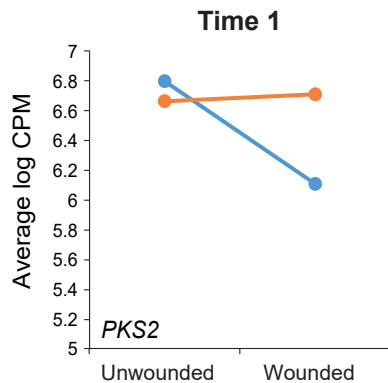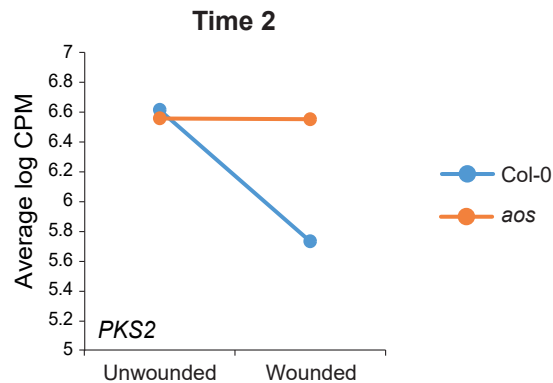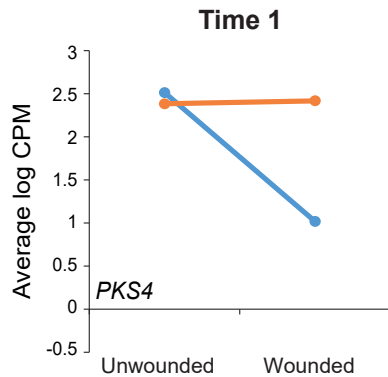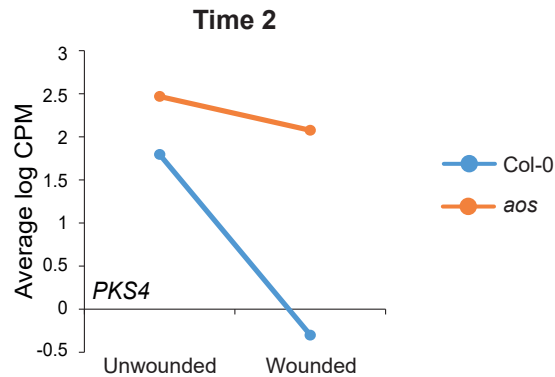

Supplement: S8 Fig — Comparison PKS2 (A, B) or PKS4 (C, D) expression in wt and aos samples under sun conditions at Time 1 (A, C) or Time 2 (B, D). Values correspond to average log CPM from the three biological replicates. (PDF) [file pgen.1010213.s012.pdf]

A

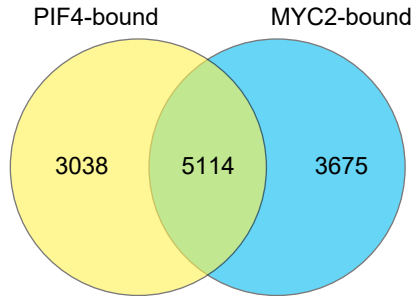

B

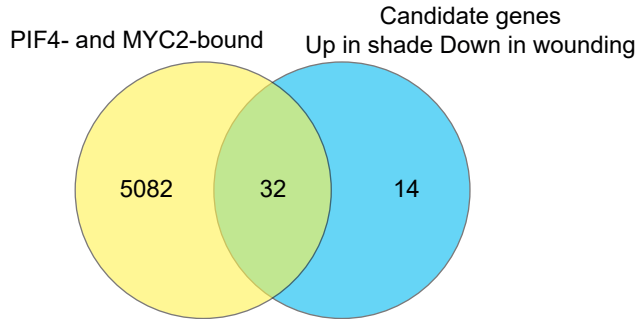

Supplement: S9 Fig — A) Venn diagram comparing PIF4-bound genes [42] to MYC2-bound genes [48] (hypergeometric test: p < 2.5 x 10^−4^). B) Venn diagram comparing genes bound by PIF4 and MYC2 (5114 genes from overlap in A) to the 46 genes from the list of candidates (S1 Table) which are up-regulated by shade and down-regulated by wounding (hypergeometric test: p < 2.5 x 10^−4^). (PDF) [file pgen.1010213.s013.pdf]
