# Supplementary material for: Shade suppresses wound-induced leaf repositioning through a mechanism involving PHYTOCHROME KINASE SUBSTRATE (PKS) genes
Source: PLoS Genet. 2022 May 27;18(5):e1010213. doi: 10.1371/journal.pgen.1010213 (PMC9197076; doi:10.1371/journal.pgen.1010213)
Supplement: S2 Fig — Petiole lengths of Col-0 with 3 different protocols of shade vs wounding treatments. Top: Schematic of the different experimental procedures. Wounds are performed individually on leaf 1, then leaf 2 and then leaf 3. The shade treatment either starts at the same time as the first wound (left), at the same time as the third wound (middle, see Fig 1A) or one day before the first wound (right). We measured petiole length of leaf 4 at the end of the treatments (day 21). Bottom: Petiole length phenotype of wt Col-0 plants (n = 12–15 plants/genotype/condition). For each protocol, a representative experiment from 3 biological replicates is presented. Different letters indicate significant differences (Tukey’s HSD test following a two-way ANOVA, P < 0.05). (PDF) [file pgen.1010213.s006.pdf]

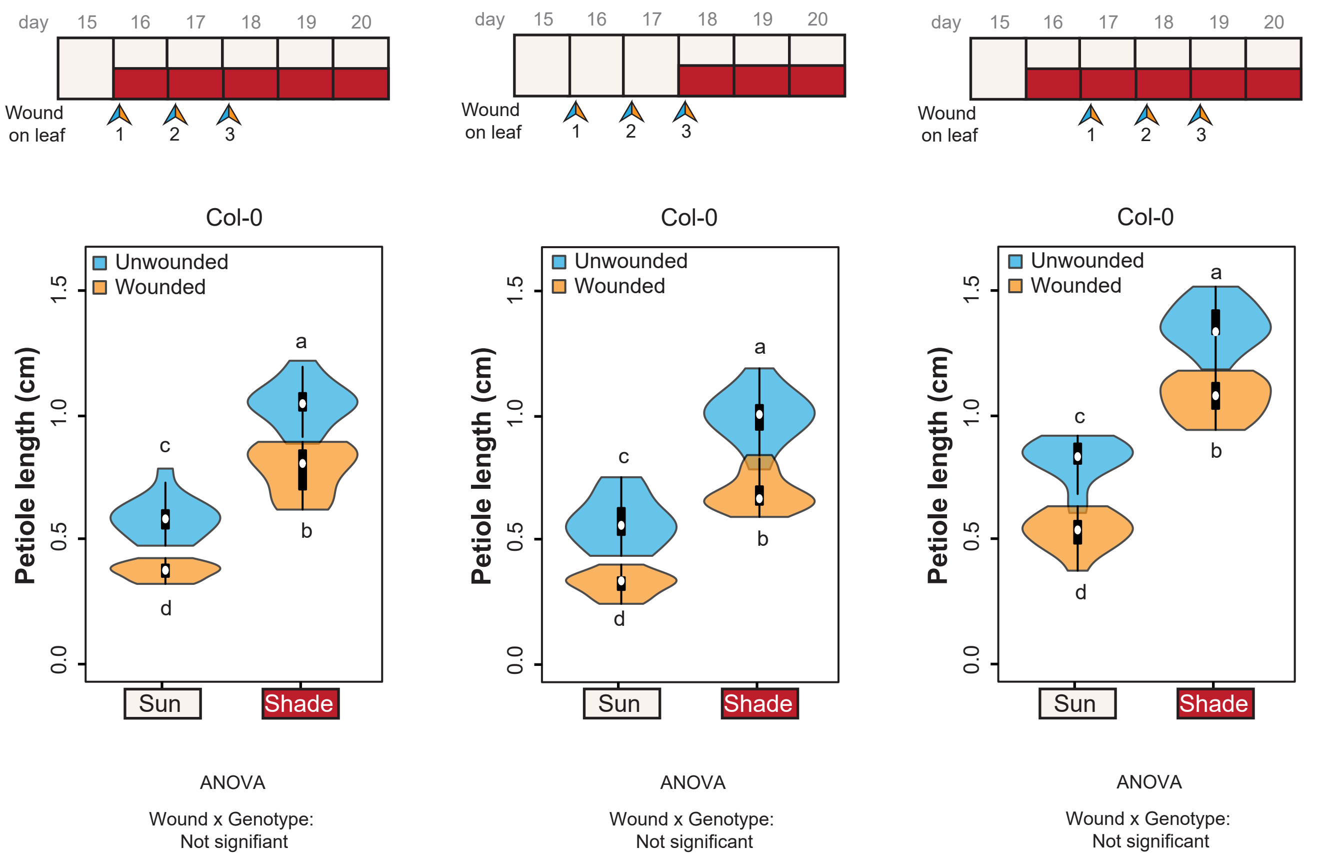
day 15 16 17 18 19 20
Wound on leaf
1 2 3
Col-0
Petiole length (cm)
0.0 0.5 1.0 1.5
Unwounded
Wounded
a b c d
Sun
Shade
ANOVA
Wound x Genotype:
Not signifiant
day 15 16 17 18 19 20
Wound on leaf
1 2 3
Col-0
Petiole length (cm)
0.0 0.5 1.0 1.5
Unwounded
Wounded
a b c d
Sun
Shade
ANOVA
Wound x Genotype:
Not significant
day 15 16 17 18 19 20
Wound on leaf
1 2 3
Col-0
Petiole length (cm)
0.0 0.5 1.0 1.5
Unwounded
Wounded
a b c d
Sun
Shade
ANOVA
Wound x Genotype:
Not significant
