# Supplementary material for: Shade suppresses wound-induced leaf repositioning through a mechanism involving PHYTOCHROME KINASE SUBSTRATE (PKS) genes
Source: PLoS Genet. 2022 May 27;18(5):e1010213. doi: 10.1371/journal.pgen.1010213 (PMC9197076; doi:10.1371/journal.pgen.1010213)
Supplement: Attachment — Submitted filename: 2022-03-11-Fiorucci-response to rev.pdf [file pgen.1010213.s014.pdf]

Response to reviewers' comments

Our answers are indicated in blue

Editor

The manuscript was fully evaluated at the editorial level and by independent peer reviewers. The reviewers recognized that this study constitutes a significant advance.

We thank the editor and reviewers for their interest in our work and their useful comments. In summary, in this revised manuscript we have now included:

- a model as figure 6.
- further support for opposite regulation of *PKS4* expression (and many other genes showing similar regulation) by MYC2 and PIF4 in figure S9.
- a new table for further statistical analysis of our physiological experiment.
- corrections to several figures based on the reviewers' comments

They, nevertheless, raised some substantial concerns about the current manuscript. While the involvement of PSKs in the interplay between wounding and shade avoidance was clearly demonstrated, this study would benefit from additional mechanistic insights into how these genes ultimately influence the hyponastic response.

The mechanism of action of PKS proteins is a difficult question to address because these proteins do not contain any protein domains of known function. Structural predictions (including the use of Alpha fold) predict that these proteins are largely intrinsically disordered. We are currently working on the mode of PKS action but will not be able to cover this in the context of this study that is aimed at studying the crosstalk between shade and wounding on leaf position. This study uncovers a role for PKS proteins for this biological response and mechanisms underlying *PKS* gene expression which plays a role in leaf positioning in different conditions (e.g. plants with altered *PKS* expression and mutants affecting *PKS* expression all show an altered hyponastic response). However, the molecular mechanism of PKS proteins goes beyond the scope of this work. Finally, we note that *PKS4* expression is induced by auxin. Given the known function of auxin in the control of hyponasty this is an interesting connection. This is now mentioned in the revised manuscript.

Some of the reviewers also thought that the conclusions concerning the regulation of PSKs by MYB were not fully supported. These conclusions could perhaps be recast as hypotheses to ensure that the readers do not misinterpret them.

We have checked the literature and found additional support for the inverse regulation of *PKS4* by JA/wounding. Our analysis reveals that in etiolated seedlings (Zander et al., 2020) *PKS4* was not identified as a gene bound by MYC2 while in rosettes (a growth stage similar to the one we used) *PKS4* was identified as a gene bound by MYC2 (Wang et al., 2019). It is therefore possible that *PKS4* regulation by MYC2 depends on the developmental stage/tissue. Further analysis of genes (including *PKS4*) potentially regulated by PIF4 and MYC2 is presented in Fig. S9.

Additional details on the statistical analyses should also be provided.

We now provide table S2 with more extensive statistical analysis as proposed by reviewer 3.

The impact of the study would be better demonstrated by proposing a model integrating the results into the current knowledge within the field.

We now present a model as proposed by reviewer 1 (Figure 6).

Reviewer #1:

Overall, this elegantly-designed study provides compelling genetic evidence showing distinct relationships between shade and wounding on leaf/petiole growth and leaf hyponasty. More importantly, the results reveal novel insight into the genetic elements required for the shade-dependent repression of the wound-induced inhibition of leaf hyponasty -- namely PIFs, auxin, PKS4/2 -- and demonstrate the function of PKS4/2 in both the positive and negative regulation of leaf hyponasty by shade and wounding, respectively. The manuscript is well written, and the figures are beautifully presented.

We thank reviewer 1 for his support and useful comments

My only suggestion is that the authors might want to elaborate the part in Discussion describing the hypothesis about the competition of PIFs and MYCs in PKS4/2 expression.

We performed additional analyses which support the idea that many genes are regulated by PIFs and MYCs (Fig. S9A). Moreover, among the genes that we identified as having an opposite regulation by shade and wounding and interaction when both signals are given together almost 70% (including *PKS4*) are putative direct targets of PIF4 and MYC2 (Fig. S9B). Interestingly in the case of *PKS4* both transcription factors appear to bind very close to each other near a PBE box. This is now further discussed in the revised manuscript.

The results in Figure S3 demonstrate that the wound-induced inhibition of leaf hyponasty depends on phytochrome B (phyB). Studies from the authors' lab and others have shown that phyB inhibits the stability and activity of PIFs. Together, the current data support the model that, in normal light conditions (without shade), phyB inhibits PIFs to allow MYCs function prominently in wounding to repress PKS4/2 expression, whereas, in shade condition, the attenuation of phyB functions enhances the amount and activity of PIFs, which can antagonize MYCs in wounding to promote PKS4/2 expression.

We now present a model (Fig. 6) to put our findings into a more global context and discuss it.

Reviewer #2:

The experiments are well planned and brilliantly carried out with a sound statistical analysis. In general, the conclusions drawn are justified except a few exceptions. For example, the last section of MYC repression of PKS2 and 4 are not convincing. The provided data is not sufficient to support this argument.

We thank the reviewer for evaluating our work and providing us with useful feedback.

We are not quite sure what the reviewer means with this particular comment. Our data on 5A shows that *PKS2* and *PKS4* expression is not repressed by wounding in the *myc234* background. This is fully consistent with expression data comparing the wild-type and *aos* on figure S8, which shows that *PKS2* and *PKS4* expression is not suppressed by wounding in *aos*. Moreover, a more extensive literature search identifies several papers showing that *PKS* gene expression is suppressed by JA application or wounding in green seedlings or leaves. Now discussed more extensively in the revised manuscript. Finally, our analysis reveals that in etiolated seedlings (Zander et al., 2020) *PKS4* was not identified as a gene bound by MYC2 while in young rosettes (a growth stage similar to the one we used) *PKS4* was identified as a gene bound by MYC2 (Wang et al., 2019). It is therefore possible that *PKS4* regulation by MYC2 depends on the developmental stage/tissue.

While the data shown here in general are well-supported, the major question remains unanswered - how do PKS proteins control the hyponasty response and how are the contrasting signals integrated? To this reviewer, more in-depth analysis on the mechanism of shade-induced suppression of wound-induced leaf positioning through PKS would have been helpful.

Our genetic data show that *PKS* genes are important for a proper leaf repositioning response when Arabidopsis integrates two signals with opposing effects of leaf position (shade and wound). Based on gene expression it is reasonable to propose that this is at least in part due to regulated *PKS* gene expression for which we provide new insight. Shade and wound lead to opposite regulation of *PKS2/4* expression that depends on PIFs, AOS and MYCs. Finally, in combined treatments the shade signal dominates *PKS* expression similar to what is observed for leaf position. Therefore, our study uncovers an interesting regulation of *PKS* gene expression and a role for PKS proteins in the integration of signals leading to opposite leaf repositioning. The importance of regulating *PKS* expression is also clear based on the leaf hyponasty phenotype of PKS over-expressing plants and mutants (*pif457* and *myc234*) in which *PKS* genes are not normally regulated (Fig. 3-5). The molecular mechanism of PKS proteins goes beyond the scope of this work. It is an interesting question that we pursue in the lab. It is also a particularly difficult one because PKS proteins do not contain any domains of known function and are predicted to be largely intrinsically disordered proteins.

What is the biological significance of the transient inhibition of leaf elevation by wounding? The authors argue that effect on leaf positioning by wounding is more than just a consequence of a general growth suppression and perhaps be of an adaptive value. This needs to be further supported. It could have helped if the role of PKS in plant defence under shade conditions were investigated.

Understanding the role of leaf position is an interesting question. Quite a number of hypotheses have been proposed regarding this response to diverse stimuli (e.g. better access unfiltered sunlight in response to shade, moving away from hot soil in response to elevated temperature). In this particular case we can only speculate that as proposed in other cases when faced with a shade and a wound (or pathogen attack) the plant prioritizes the shade response (see e.g. Fernandez-Milmanda & Ballaré, 2021 in TIPS). This is stated in our discussion. As indicated in our manuscript the scope of our work is to study the growth and leaf movement response and not the defense response which was already the scope of many publications. This is why the implications of our findings are simply discussed.

To this reviewer, while this is an interesting and well executed study, the findings are rather incremental and stops short of making a significant advance.

We respectfully disagree with the reviewer. The fact that combined shade and wound treatments affects growth and leaf position differently is novel. So is the identification of a role of *PKS* genes for the coordinated response and the complex regulation of *PKS* gene regulation.

Reviewer #3: Review of Fiorucci et al for PLoSG

This manuscript explore the relationship between shade and defense signaling in the control of plant morphology. Although the effects of shade and defense signaling interactions on defense responses have been explored in a number of prior publications, the effect of these interactions on growth and morphology is much less explored. Thus, this manuscript is novel and is a significant contribution to the field. This manuscript describes the molecular-genetic mechanisms underlying the effect of shade/defense interactions on leaf angle, and reveals that genes in the PKS family are an important intersection point for these two pathways. The manuscript is well written and conclusions are well supported. There are some issues that need to be addressed before considering publication.

We thank the reviewer for his/her interest in our work, careful reviewing and helpful suggestions

Major:

1) The authors report p-values for the interaction term in two-way ANOVA tests but not in three-way ANOVA tests (e.g. genotype:light:wounding for fig 1BC, 3AB, etc). In theory the three-way interaction term from an ANOVA or linear regression would be the best test for whether or not a mutant affects the light:wounding interaction, but I acknowledge that it can be difficult to achieve enough power to adequately test this term in practice, potentially leading to false negatives. Another approach might be to fit separate two-way linear regressions for WT and mutant, obtain 95% confidence intervals on the interaction term and determine if they overlap or not. Current conclusions are based on (I think) Tukey's HSD but this is not completely clear. If the authors stick with the Tukey HSD approach it would be helpful if they explained how they are interpreting these tests to arrive at their conclusions (along the lines of "shade caused a similar increase in leaf angle in unwounded Col-0 and pks2pks4 (Tukey HSD group a) but this effect was reduced in the pks2pks4 double mutant (Tukey HSD group b for Col-0 vs group c for pks2pks4)". They is probably a more elegant way to write this, but hopefully that gets the idea across. The reason that the regression or ANOVA approach is favored over the Tukey HSD is that the term that we want to test is related to differences in the wounding response, which is only indirectly addressed by the Tukey approach. In the end I am OK with the Tukey approach if it is better explained, but I suggest that the authors at least consider the other approaches I have outlined if they have not already. On a related note, the figure legends and methods state that a three-way ANOVA was performed. The methods should describe the formula for that (were all two and three way interaction terms used?)

To address this comment we performed additional statistical analyses and included a new table that contains the results. Indeed, in some cases a mutant shows a different response from the wild type in combined treatments (shade + wound), however the interaction between both treatments is not significantly different in both genotypes. This is for example the case for *pks4pks2*, while in *pks1pks2pks3pks4* both the response and the interaction between treatments differ between the mutant and the wild type. As suggested by the reviewer this could be due to a lack of statistical

power because it is difficult to compare enough plants from multiple genotypes across 4 different conditions in an experiment. We discuss this more carefully in the revised ms.

2) The authors conclude that the transcriptional response to wounding is more rapid than the response to shade (line 218) but I do not think that the data supports this, because the first wounding treatment is 2 days earlier than the shade treatment (Fig 1A).

This is an excellent comment, we have changed the text accordingly

Minor:

Lines 152-183. This is a long paragraph that is a bit difficult to follow, especially because the authors go back and forth between the different responses that they are measuring are not always clear. For example, lines 167-168 “We concluded that there is no interaction shade and wounding…” this is for petioles but that is not indicated in this sentence or the one above. And in this section of the paragraph petiole length is discussed before leaf angle even though they are in the opposite order in the figure. Anyway: the authors should consider whether this section can be organized in a more clear manner; it is easy for the reader to get lost.

We have modified the text.

Fig 2B: the order of the timepoints in the heat map is reversed that from 2A (and from the progression of time) and should be flipped. In the worst case the heat map could be flipped and relabeled “by hand” (outside of R) if it is not possible to have R present time 1 on the left.

Thank you for the suggestion, this was modified for clarity.

Fig S4B: are the genotype labels wrong? If not, why does aos have a stronger response than Col on the heatmap?

Thank you for spotting this mistake which was corrected.

Fig 3B y-axis label (left plot) is “Petiole angle” but elsewhere “Leaf angle” is used.

This is not a mistake and is explained in M&M and now also in the figure legend. *pks1pks2pks3pks4* mutants have downwards curled leaf blades (see Legris et al., 2021 in Plant Physiology). This potentially affects the measured leaf position if it were done as in the other experiments because both the petiole angle and the blade angle influence the final leaf position if one considers the leaf tip only (see Dornbush et al., 2014 in Plant Cell). Therefore in this particular experiment we specifically measured the petiole angle.

Fig 4B are “peak” and “control” mislabeled in the bar plot? Also PKS4 doesn’t have solid evidence for induction by shade. Could say marginal in text.

Thank you for spotting this mistake which was corrected.

qPCR methods: although a citation to a previous paper is given, it would be nice if a sentence or two

could be added about the analysis (is this delta-delta CT?) so that the curious does not have to flip back to the previous paper.

We have expanded the explanation on this in M&M.
